# Longitudinal Changes in Left Ventricular Geometry After Kidney Transplantation and Their Implications on Cardiovascular Risk

**DOI:** 10.1016/j.xkme.2025.101201

**Published:** 2025-12-11

**Authors:** Dong-Hyuk Cho, Jun Gyo Gwon, Jimi Choi, Cheol Woong Jung, Tai Yeon Koo, Se Won Oh, Sang-Kyung Jo, Kyo Won Lee, Kyu Ha Huh, Han Ro, Seung-Yeup Han, Jang-Hee Cho, Sik Lee, Jaeseok Yang, Seong-Mi Park, Myung-Gyu Kim

**Affiliations:** 1 Division of Cardiology, Department of Internal Medicine, Korea University Medicine, Seoul, Republic of Korea; 2 Division of Vascular Surgery, Department of Surgery, Asan Medical Center, University of Ulsan College of Medicine, Seoul, Republic of Korea; 3 Division of Endocrinology and Metabolism, Department of Internal Medicine, Korea University Medicine, Seoul, Republic of Korea; 4 Department of Transplantation and Vascular Surgery, Korea University College of Medicine, Seoul, Republic of Korea; 5 Department of Internal Medicine, Korea University College of Medicine, Seoul, Republic of Korea; 6 Department of Surgery, Seoul Samsung Medical Center, Sungkyunkwan University, Seoul, Republic of Korea; 7 Department of Surgery, Yonsei University College of Medicine, Seoul, Republic of Korea; 8 Department of Internal Medicine, Gachon University Gil Hospital, Incheon, Republic of Korea; 9 Department of Internal Medicine, Keimyung University College of Medicine, Daegu, Republic of Korea; 10 Department of Internal Medicine, Kyungpook National University Hospital, Daegu, Republic of Korea; 11 Department of Internal Medicine, Chonbuk National University Hospital, Jeonju, Republic of Korea; 12 Department of Internal Medicine, Yonsei University, Seoul, Republic of Korea

**Keywords:** Heart ventricle, cardiovascular disease, triglycerides, hemodynamics, kidney transplantation

## Abstract

**Rationale & Objective:**

Kidney transplantation (KT) alleviates the hemodynamic burden in chronic kidney disease on dialysis. However, cardiovascular disease remains the leading cause of death after KT. This study evaluated the metabolic and hemodynamic burden and its impact on myocardial remodeling and clinical outcomes after KT.

**Study Design:**

Multicenter observational prospective cohort study.

**Setting & Participants:**

600 patients who underwent echocardiography before and 3 years after KT from 8 university hospitals in Korea.

**Predictors:**

Changes in metabolic parameters (glycosylated hemoglobin [HbA_1C_] and triglyceride [TG] levels) and hemodynamic parameters (hemoglobin [Hb] and systolic blood pressure [SBP]) from baseline to 3 years after KT.

**Outcomes:**

Primary outcome was major adverse cardiovascular events (MACE). Secondary outcomes included left ventricular geometry changes.

**Analytical Approach:**

Cox proportional hazards models were used to assess the association between echocardiographic changes and MACE.

**Results:**

TG and HbA_1c_ levels increased 3 years after KT; however, Hb levels and SBP improved (*P* < 0.05). Left ventricular end-diastolic dimension also improved for 3 years (*P* < 0.05). Nevertheless, the relative wall thickness (RWT) increased from 0.39 ± 0.07-0.41 ± 0.07. Changes in hemodynamic parameters (Hb level and SBP) were associated with a change in the left ventricular end-diastolic dimension, whereas changes in metabolic parameters (HbA_1c_ and TG levels) were associated with a change in RWT (*P* < 0.05). During the 5-year follow-up, 30 MACE occurred, and an increase in RWT independently predicted MACE occurrence (hazard ratio, 2.20; 95% confidence interval, 1.21-3.99; *P* < 0.01).

**Limitations:**

Only patients with baseline and follow-up echocardiography were included, potentially introducing selection bias.

**Conclusions:**

Hemodynamic improvements are associated with decreased left ventricular size; better metabolic control is associated with greater wall thickness improvement. RWT increases predicted MACE. Optimizing metabolic control to promote balanced left ventricular improvement could enhance cardiovascular outcomes in patients receiving KTs.

## Plain-Language Summary

Patients receiving a kidney transplant often develop cardiovascular complications despite successful transplantation. To examine how cardiac structure changes posttransplant and their impact on future cardiovascular risk, we analyzed 600 patients in the prospective multicenter cohort, performing echocardiography before and 3 years after transplant. Overall, cardiac structure improved following transplantation, but some developed increased relative wall thickness. Better blood pressure and anemia control showed greater improvement in cardiac chamber size, whereas better glycemic and lipid control achieved superior wall thickness improvement. Importantly, patients with increased relative wall thickness faced twice the risk of major adverse cardiovascular events, including myocardial infarction and stroke. These findings highlight the importance of optimizing metabolic control to promote balanced cardiac remodeling and reduce cardiovascular risk after transplantation.

## Introduction

Kidney transplantation (KT) significantly improves the patient's quality of life by alleviating uremia, anemia, electrolyte imbalance, and fluid overload. However, chronic cardiovascular alterations, such as atherosclerosis, vascular calcification, and fibrosis, that are associated with chronic kidney disease (CKD) may not improve after transplantation. Furthermore, metabolic conditions, such as dyslipidemia, obesity, insulin resistance, and posttransplant diabetes mellitus, owing to the use of immunosuppressants, anabolic energy metabolism, increased appetite, and weight gain after KT, can negatively affect vascular health. As a result, cardiovascular diseases (CVDs) remain the leading cause of morbidity and mortality among patients receiving KTs after the transplantation.^1^, ^2^, ^3^

Echocardiographic imaging of structural and functional changes can provide valuable information regarding the risk of CVDs. Several studies have reported longitudinal changes in echocardiographic parameters after KT. The results of cardiac remodeling are inconsistent, although KT can improve cardiac dysfunction and some structural changes in patients with CKD. Cardiac structural abnormalities, including abnormalities in the interventricular septum, left ventricular (LV) end-diastolic dimension (LVEDD), and LV mass, have been reported to normalize within 7 years after KT in pediatric patients with a relatively short duration of CKD.^4^ However, other studies have demonstrated that cardiac function can improve after KT without leading to major changes in ventricular morphological features.^5^ Additionally, KT has been reported to be associated with improved cardiac function with little or no change in LV geometry.^6^ Differences in cardiac remodeling following KT may influence the onset or exacerbation of CVDs, subsequently affecting KT outcomes differently; however, our understanding of these details is limited.

This large prospective cohort study involving patients receiving KTs aimed to investigate (1) longitudinal changes in eccentric and concentric myocardial remodeling and cardiac function, (2) clinical factors associated with these longitudinal changes, and (3) the impact of these longitudinal changes on CVDs and KT outcomes.

## Methods

### Database and Study Design

This observational, prospective cohort study was conducted at 8 centers in the Republic of Korea. A total of 1,080 patients who underwent KT were enrolled in the Korean Cohort Study for Outcome in Patients with KT (KNOW-KT) from July 2012 to August 2016, and 600 patients who underwent 2 conventional echocardiographies at baseline and follow-up were included in the present study. Detailed protocols of the KNOW-KT registry were previously described.^7^ Briefly, consecutive patients aged >18 years old, who received KT from 8 university hospitals, were enrolled. Patients with simultaneous multiple organ transplantation, en bloc KT, liver cirrhosis, or interstitial lung disease were excluded. Patients were followed up for 5 years or until their death, graft failure, or dropout. KNOW-KT was approved by the Institutional Review Committee of each participating center, and written informed consent was obtained from all patients. The procedures performed in this study were in accordance with the institutional guidelines and regulations.

### Clinical Variables

Baseline clinical variables, such as demographic data, underlying medical disease history, medications, information about renal replacement therapy, and smoking history, were obtained during the pretransplant screening period. Frailty was assessed using the age-adjusted Charlson comorbidity index.^8^ Baseline laboratory tests were performed before KT; however, serum urea nitrogen and creatinine values were evaluated 4 weeks later, after KT, because of their variability in the immediate postdialysis period before KT. All the patients fasted for at least 8 h before blood sampling. The levels of hemoglobin (Hb), creatinine, glycosylated Hb (HbA_1c_), total cholesterol, triglycerides (TGs), low-density lipoprotein cholesterol (LDL-C), and high-density lipoprotein cholesterol (HDL-C) were measured using blood tests. The estimated glomerular filtration rate was calculated using the CKD-EPI equation. All variables and values collected at baseline admission and every follow-up year were recorded in an electronic case report form.

### Echocardiographic Parameters

All patients underwent conventional echocardiographies using a commercially available echocardiographic system to assess LV geometry and function. Chamber quantification was performed using 2-dimensional or M-mode imaging techniques following the guidelines of the American Society of Echocardiography and the European Association of Cardiovascular Imaging.^9^ LV geometries were evaluated by measuring LVEDD, the interventricular septum, and the posterior wall thickness. Relative wall thickness (RWT) was calculated as (2 × posterior wall thickness)/LVEDD, and LV mass index (LVMI) was calculated using formulas recommended by the American Society of Echocardiography.^10^ LV ejection fraction (LVEF) was calculated using the biplane Simpson method from apical 4- and 2-chamber views.

### Clinical Outcomes

**Figure 1:**
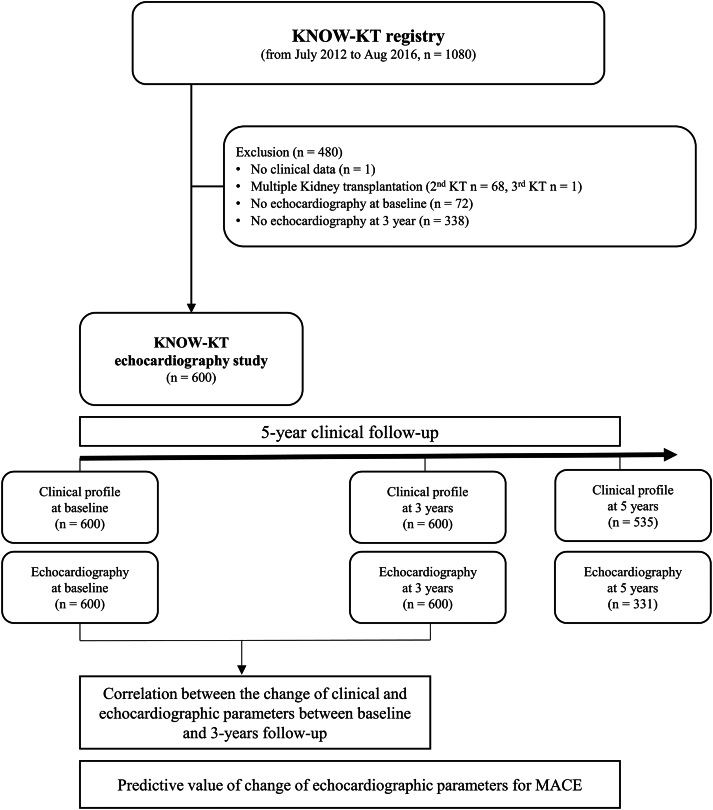
Study algorithm. Abbreviations: KNOW-KT, Korean Cohort Study for Outcome in Patients with KT; KT, kidney transplantation; MACE, major adverse cardiovascular events.

The primary outcome of the study was major adverse cardiovascular events (MACE), which were defined as a composite of new-onset coronary artery disease, cerebrovascular disease, peripheral arterial disease, and cardiovascular mortality. The diagnosis was confirmed using the KNOW-KT registry survey conducted at each center. The specific diagnosis for each disease group was rechecked to ensure diagnostic accuracy. Coronary artery disease included angina pectoris, myocardial infarction, and silent myocardial ischemia, whereas cerebrovascular disease included acute neurogenic injury resulting from ischemic cerebral infarction. Hemorrhagic cerebrovascular disease was excluded because it may have a different pathophysiology compared with ischemic cerebrovascular disease, which is primarily caused by atherosclerosis. Follow-up was conducted for a period of 5 years after KT (Fig 1).

### Statistical Analysis

All variables collected were presented as frequencies (percentages) for categorical variables or as mean ± standard deviation or median (interquartile range) for continuous variables according to the distribution of the variables. The changes in clinical parameters or cardiac indices over time were analyzed using a paired *t* test or the Wilcoxon signed-rank test for continuous variables and the McNemar’s test for binary variables. The linear correlation between changes from baseline to 3 years in clinical and echocardiographic variables was determined using Pearson’s correlation analysis. We estimated the cumulative incidence of the outcomes according to quartile groups of key echocardiographic variables (LVEF, LVMI, LVEDD, and RWT) using the Kaplan-Meier curves and performing the log-rank test to evaluate the effect of these changes on the clinical outcomes. The effect of changes from baseline to 3 years in echocardiographic parameters on the MACE was evaluated using the multivariable Cox proportional regression models, with the results presented as hazard ratios per 1 standard deviation of changes and 95% confidence intervals. Adjusted variables included in the model were age, sex, value of baseline parameters, and statistically significant covariates based on univariable analysis of age, sex, donor type, donor age, diabetes, hypertension, dyslipidemia, smoking, dialysis duration, use of tacrolimus, changes from baseline, and 3-year in clinical parameters (body mass index [BMI, kg/m^2^], waist-to-hip ratio, Hb level, estimated glomerular filtration rate, HbA_1c_ level, SBP, DBP, LDL-C level, TG level, HDL-C level, and TG/HDL-C ratio change) for MACE (Table S1). All statistical analyses were performed using SAS 9.4 software (SAS Institute), and a *P* value < 0.05 was considered statistically significant.

## Results

### Baseline Characteristics of the Study Population

The mean age of the study population was 46.2 ± 11.4 years, and 62% of patients were men. Living- and deceased-donor transplantations were performed in 512 (85.3%) and 88 (14.7%) patients, respectively. The causes of end-stage kidney disease were diabetes, hypertension, glomerulonephritis, polycystic kidney disease, and unknown in 128 (21.3%), 122 (20.3%), 188 (31.3%), 34 (5.7%), and 96 (16.0%) patients, respectively. Patients received steroids (600, 100%) and tacrolimus (571, 95.2%) as maintenance immunosuppressants after KT. Table S2 displays the patient characteristics.

### Post-KT Longitudinal Changes in Clinical Parameters and Cardiac Structure and Function

The BMI increased significantly from 23.0 ± 3.4-23.4 ± 3.2 kg/m^2^ 3 years after KT (*P* < 0.001). Hb levels significantly increased from 10.5 ± 1.6-14.0 ± 1.9 g/dL (*P* < 0.001), and SBP and DBP decreased from 137.4 ± 19.2 and 83.5 ± 12.6-127.2 ± 14.5 and 78.2 ± 10.8 mmHg, respectively (both *P* < 0.001). However, the prevalence of diabetes mellitus and dyslipidemia increased from 25.7%-38.7% (*P* < 0.001) and 53.7%-86.3% (P < 0.001), representing 13% and 32.6% absolute increases in new-onset diabetes mellitus and dyslipidemia, respectively. HbA1c, LDL-C, and TG levels also increased from 5.6% ± 0.8%-6.3% ± 1.3% (*P* < 0.001), 83.8 ± 30.6-95.0 ± 29.7 mg/dL (*P* < 0.001), and 128.8 ± 85.7-141.1 ± 93.8 mg/dL (*P* = 0.002), respectively. Similar changes were observed after 5 years of KT (Table 1).

**Table 1:** Changes in Clinical Parameters Between Baseline and Follow-up

Mean ± SD or n (%)	Baseline (N = 600)	At 3 y (N = 600)	At 5 y (N = 535)	*P*^a^	*P*^b^
BMI (kg/m^2^)	23.0 ± 3.4	23.4 ± 3.2	23.6 ± 3.2	< 0.001	< 0.001
WHR	0.89 ± 0.06	0.88 ± 0.05	0.89 ± 0.06	0.435	0.324
SBP (mmHg)	137.4 ± 19.2	127.2 ± 14.5	127.2 ± 13.5	< 0.001	< 0.001
DBP (mmHg)	83.5 ± 12.6	78.2 ± 10.8	78.0 ± 10.6	< 0.001	< 0.001
Heart rate (bpm)	77.2 ± 12.6	77.2 ± 12.6	78.4 ± 12.2	0.713	0.156
Comorbid conditions					
DM	154 (25.7)	232 (38.7)	217 (40.6)	< 0.001	< 0.001
HTN	553 (92.2)	570 (95.0)	511 (95.5)	0.045	< 0.001
Dyslipidemia	322 (53.7)	518 (86.3)	440 (82.4)	< 0.001	< 0.001
CAD	40 (6.7)	53 (8.8)	49 (9.2)	0.161	< 0.001
Stroke	23 (3.8)	29 (4.8)	24 (4.5)	0.395	0.045
Heart failure	13 (2.2)	14 (2.3)	13 (2.4)	0.846	0.317
Arrhythmia	7 (1.2)	14 (2.3)	13 (2.4)	0.123	0.014
Liver disease	36 (6.0)	46 (7.7)	42 (7.9)	0.253	0.001
Chronic lung disease	6 (1.0)	9 (1.5)	10 (1.9)	0.436	0.045
Cancer	23 (3.8)	35 (5.8)	38 (6.2)	0.106	< 0.001
Modified Charlson comorbidity index, median (IQR)	3 (2–4)	1 (0–3)	1 (0–3)	< 0.001	< 0.001
Laboratory findings					
Hemoglobin level (g/dL)	10.5 ± 1.6	14.0 ± 1.9	13.8 ± 2.0	< 0.001	< 0.001
Creatinine level (mg/dL)	1.18 ± 0.44	1.21 ± 0.84	1.19 ± 0.58	0.343	0.333
eGFR (mL/min/1.732m^2^)	66.4 ± 19.4	66.6 ± 20.4	66.1 ± 21.1	0.860	0.414
HbA1C level (%)	5.6 ± 0.8	6.3 ± 1.3	6.4 ± 1.3	< 0.001	< 0.001
LDL-C level (mg/dL)	83.8 ± 30.6	95.0 ± 29.7	92.2 ± 28.9	< 0.001	< 0.001
TG (mg/dL)	128.8 ± 85.7	141.1 ± 93.8	145.6 ± 108.8	0.002	< 0.001
HDL-C level (mg/dL)	45.1 ± 16.0	59.9 ± 18.0	60.2 ± 18.0	< 0.001	< 0.001
TG/HDL-C ratio	3.50 ± 3.49	2.85 ± 3.33	2.94 ± 3.91	< 0.001	0.001
HTN medications	540 (90.5)	285 (57.9)	268 (66.8)	< 0.001	< 0.001
RAS blockade	312 (52.3)	123 (25.0)	97 (24.2)	< 0.001	< 0.001
Diuretics	152 (25.5)	20 (4.1)	6 (1.5)	< 0.001	< 0.001
β-blocker	278 (46.6)	194 (39.4)	129 (32.2)	0.018	< 0.001
CCB	394 (66.0)	164 (33.3)	185 (46.1)	< 0.001	< 0.001
Antithrombotics	481 (80.2)	90 (15.0)	71 (17.7)	< 0.001	< 0.001
Lipid-lowering agents	191 (31.8)	374 (62.3)	268 (66.8)	< 0.001	< 0.001
DM medications	142 (23.7)	220 (36.7)	155 (38.7)	< 0.001	< 0.001
Insulin	80 (13.3)	120 (20.0)	80 (20.0)	0.002	0.005
OHA	82 (13.7)	178 (29.7)	125 (31.2)	< 0.001	< 0.001
Immunosuppressants					
Tacrolimus	571 (95.2)	577 (96.2)	381 (95.0)	0.395	0.912
Cyclosporin	27 (4.5)	18 (3.0)	15 (3.7)	0.172	0.557
Mycophenolate mofetil	317 (52.8)	295 (49.2)	196 (48.9)	0.204	0.220
Myfortic acid	181 (30.2)	154 (25.7)	89 (22.2)	0.082	0.005
Bredinine	12 (2.0)	55 (9.2)	43 (10.7)	< 0.001	< 0.001
Sirolimus	31 (5.2)	44 (7.3)	39 (9.7)	0.121	0.006
Everolimus	15 (2.5)	1 (0.2)	1 (0.2)	< 0.001	0.005
Steroids	600 (100)	541 (90.2)	362 (90.3)	< 0.001	< 0.001

Abbreviations: BMI, body mass index; CAD, coronary artery disease; CCB, calcium channel blocker; DM, diabetes mellitus; eGFR, estimated glomerular filtration rate; HbA_1C_, glycosylated hemoglobin; HDL-C, low-density lipoprotein cholesterol; HTN, hypertension; IQR, interquartile range; LDL-C, low-density lipoprotein cholesterol; OHA, oral hypoglycemic agents; RAS, renin-angiotensin system; SD, standard deviation; TG, triglyceride; WHR, waist-to-hip ratio.

a *P*-value for comparison between baseline and 3 years.

b *P*-value for comparison between baseline and 5 years.

**Figure 2:**
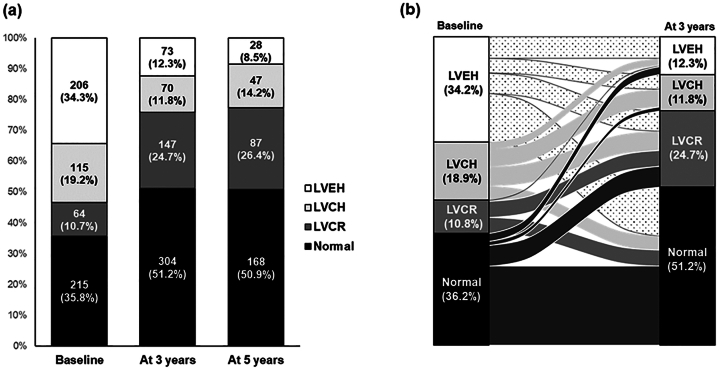
Changes in LV geometry after kidney transplantation. (A) Distribution of LV geometry at baseline and after 3 and 5 years. (B) Longitudinal changes in LV geometry at baseline and 3-year follow-up. Abbreviations: LV, left ventricular; LVEH, left ventricular eccentric hypertrophy; LVCH, left ventricular concentric hypertrophy; LVCR, left ventricular concentric remodeling.

All 600 patients underwent conventional echocardiographies at baseline and 3 years, but only 331 patients underwent echocardiographies at 5 years. Baseline and follow-up echocardiographies revealed changes in LVEDD, LVEF, RWT, and LVMI at 3 and 5 years after KT. The LV chamber size, represented using LVEDD, improved significantly over 3 years (51.2 ± 5.6-47.1 ± 4.8 mm, *P* < 0.001). LV wall thickness and LVMI decreased significantly (LVSWT, 10.0 ± 1.6-9.7 ± 1.5 mm, *P* < 0.001; LV posterior wall thickness, 10.0 ± 1.6-9.5 ± 1.5 mm, *P* < 0.001; LVMI, 113.4 ± 31.8-94.4 ± 23.4 g/m^2^, *P* < 0.001). LV contractility, evaluated using LVEF, improved significantly from 61.4% ± 7.9%-64.8% ± 6.0% (*P* < 0.001). However, the parameter of LV concentricity, RWT, increased from 0.39 ± 0.07-0.41 ± 0.07 mm, reflecting that chamber size improved more than wall thickness in 3 years after KT (*P* < 0.001) (Table S3). Figure 2 demonstrates the longitudinal change in LV geometry after KT. The prevalence of normal LV geometry and LV concentric remodeling increased; however, both LV concentric and eccentric hypertrophy decreased.

### Clinical Factors Associated With Structural and Functional Cardiac Changes

**Figure 3:**
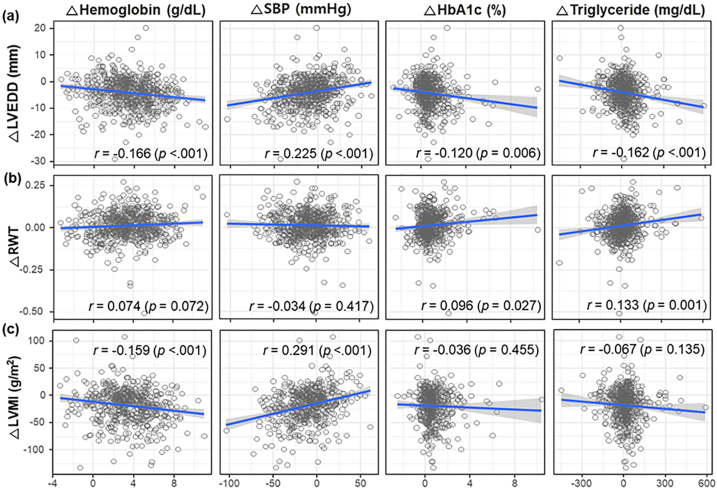
Correlation between changes in clinical and echocardiographic parameters from baseline to 3 years. The correlation of clinical parameters, including hemoglobin level, SBP, HbA_1c_, and triglyceride, and echocardiographic parameters, including (a) LVEDD, (b) RWT, and (c) LVMI, was demonstrated. Abbreviations: HbA_1c_, glycosylated hemoglobin; LVEDD, left ventricular end-diastolic dimension; LVMI, left ventricular mass index; SBP, systolic blood pressure; RWT, relative wall thickness.

Clinical factors associated with structural and functional cardiac changes were analyzed using correlation analysis of changes in echocardiographic and clinical parameters. These results showed that a decrease in LVEDD was associated with an increase in Hb levels (r = −0.166, *P* < 0.001), a decrease in SBP (r = 0.225, *P* < 0.001), an increase in HbA_1c_ level (r = −0.120, *P* = 0.006), and an increase in TG level (r = −0.162, P = 0.001). Additionally, an increase in RWT was correlated with an increase in HbA_1c_ level (r = 0.096, *P* = 0.027) and TG level (r = 0.133, *P* = 0.001). Moreover, a decrease in LVMI was associated with an increase in Hb level (r = −0.159, *P* < 0.001) and a decrease in SBP (r = 0.291, *P* < 0.001). Furthermore, an increase in LVEF was associated with an improvement in estimated glomerular filtration rate (r = 0.105, *P* = 0.001) (Fig 3 and Table S4).

### Impact of Structural and Functional Cardiac Changes on KT Outcomes

During the 5-year follow-up period, 30 MACE occurred (cumulative incidence: 5%). The breakdown of MACE included coronary heart disease (n=23, 3.8%), stroke (n=7, 1.2%), and cardiovascular death (n=5, 0.8%). The impact of echocardiographic parameters on KT outcomes was evaluated. However, no baseline echocardiographic parameters predicted the future development of MACE (Table S5). Among the parameters of change of echocardiographic parameters in the univariate analysis, only the change in RWT was related to the new development of MACE (log-rank *P* = 0.037), whereas changes in LVEF, LVMI, and LVEDD were not (Table S6).

**Figure 4:**
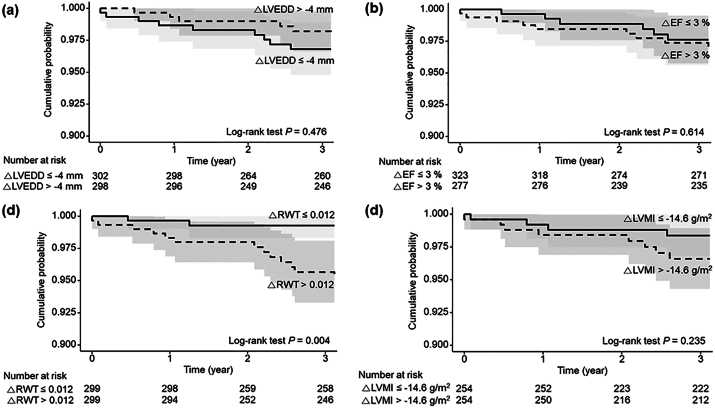
Kaplan-Meier curves for study outcomes according to median value of change in (a) LVEDD, (b) EF, (c) RWT, (d) LVMI. Abbreviations: EF, ejection fraction; LVEDD, left ventricular end-diastolic dimension; LVMI, left ventricular mass index; RWT, relative wall thickness.

In multivariate analysis, only an increase in RWT, but not other echocardiographic parameters, independently predicted the occurrence of MACE after adjusting for age, sex, and other baseline clinical and echocardiographic parameters (hazard ratio, 2.20; 95% confidence interval, 1.21-3.99; *P* < 0.01) (Table 2). Patients with increased RWT showed a statistically significantly higher rate of MACE for up to 5 years following the KT (log-rank *P* = 0.004). The Kaplan-Meier curves for the changes in key echocardiographic parameters are shown in Fig 4.

**Table 2:** Impact of Changes in Echocardiographic Parameters on the Major Adverse Cardiovascular Events

Parameters	Univariable analysis		Multivariable analysis^a^	
	HR (95% CI)	*P*	HR (95% CI)	*P*
LVEF change per 1SD	0.86 (0.51, 1.45)	0.565	0.64 (0.32, 1.29)	0.213
LVMI change per 1SD	1.49 (0.84, 2.66)	0.176	1.53 (0.75, 3.14)	0.246
LVEDD change per 1SD	0.84 (0.51, 1.37)	0.474	0.63 (0.33, 1.23)	0.178
RWT change per 1SD	2.11 (1.27, 3.50)	0.004	2.20 (1.21, 3.99)	0.010

Abbreviations: CI, confidence interval; HR, hazard ratio; LVEDD, left ventricular end-diastolic dimension; LVEF, left ventricular ejection fraction; LVMI, left ventricular mass index; RWT, relative wall thickness; SD, standard deviation.

a adjusted by age, sex, and baseline clinical and echocardiographic parameters.

## Discussion

KT recipients often display a complex CVD burden. Although preexisting CVD risk factors, such as volume overload, elevated blood pressure, anemia, and uremia, may improve after KT, other factors, such as dyslipidemia, glucose intolerance, and diabetes, can persist or even worsen caused to posttransplant alterations, including the use of immunosuppressant drugs, increased BMI, and posttransplant complications.^3^^,^^11^ Despite a recent decline in CVD-related deaths among transplant patients, it remains the leading cause of death in this population.^1^^,^^2^ Assessing the cardiovascular risk before KT, correcting modifiable abnormalities, and properly managing the dynamic cardiovascular risks that can arise posttransplant are important to reduce CVD risk.

Echocardiography can be a useful tool for assessing cardiac function and identifying patients at risk of CVD. Pretransplant LV geometric parameters can also be linked to cardiovascular risk, with some studies showing that concentric hypertrophy before transplant is associated with MACE after transplant.^12^ However, the relationship between LV geometry and transplant outcomes is complex and not fully understood. Successful KT leads to LV hypertrophy regression, and the longitudinal changes in LV geometry may affect the occurrence of CVDs differently. A recent multicenter study demonstrated that persistent LV hypertrophy was independently associated with allograft failure but not with cardiovascular morbidity or mortality.^13^ In another study, improvement in LVEF after KT, rather than structural changes, was associated with better long-term prognosis in patients with LV systolic dysfunction.^6^ However, most studies had retrospective designs with a small number of patients and different cohort structures in terms of race, comorbid condition, and CVDs, showing inconsistent results for the effect of LV geometry on KT outcomes. Conversely, this large prospective multicenter cohort study successfully determined the changes in LV remodeling during the KT and their effect on long-term clinical outcomes.

In this study, the prevalence of eccentric hypertrophy decreased, whereas concentric remodeling increased in the first 3 years after transplant. Improved anemia and blood pressure were strongly associated with a decrease in LVMI and eccentricity after KT, consistent with previous studies demonstrating that post-KT Hb levels and blood pressure are important predictors of LVEF or LVMI improvements.^4^^,^^6^ Additionally, increases in HbA_1c_ and TG levels were associated with increased RWT, suggesting that posttransplant metabolic changes may contribute to concentric hypertrophy. However, it is important to note that all individual cardiac structural parameters (LVEDD, wall thickness, and LVMI) improved after KT. The increase in RWT should therefore be interpreted as reflecting the relatively greater improvement in LV chamber size compared with wall thickness, rather than true worsening of cardiac geometry. In this context, a more mechanistically accurate interpretation would be that better control of diabetes and hyperlipidemia might be associated with more complete improvement in LV wall thickness parameters, leading to more balanced cardiac remodeling. This enhanced structural improvement could potentially translate to further reduction in MACE occurrence in patients receiving a kidney transplant because our results demonstrate that incomplete LV remodeling (reflected by increased RWT) independently predicts adverse cardiovascular outcomes.

A previous study found that myocardial TG level independently predicted myocardial concentricity in patients with diabetes as a potential driver of concentric remodeling; however, blood pressure was not correlated with concentric remodeling.^14^ Although the mechanism of LV concentric remodeling remains unclear, it is suggested to be caused by excessive accumulation of TGs in myocytes. Several animal and clinical studies have demonstrated an association between lipotoxicity and concentric myocardial changes.^15^, ^16^, ^17^, ^18^ Notably, glucose intolerance has also been associated with increased LV posterior wall thickness, interventricular septum, RWT, and impaired function, particularly diastolic function.^19^

In the posttransplant setting, weight gain and insulin resistance cause hyperinsulinemia and increased lipolysis, leading to increased free fatty acid uptake in the liver. Steroids also increase free fatty acid synthase and acetyl coenzyme A carboxylase levels, resulting in the overproduction of TGs and very LDL-Cs.^20^, ^21^, ^22^, ^23^ After KT, metabolic alterations, such as glucose intolerance, hyperlipidemia, and obesity, may contribute to cardiac steatosis, leading to concentric remodeling and hypertrophy. The counterintuitive association between metabolic parameters (HbA_1c_ and TG levels) and LVEDD improvement likely reflects the complex interplay between hemodynamic recovery and metabolic burden in the posttransplant setting. Although hemodynamic factors remained the primary drivers of LVEDD changes, metabolic control appeared to modulate the extent of cardiac structural recovery, suggesting that patients with better metabolic management might achieve more complete cardiac remodeling.

In our study, we also found an increase in the BMI, together with an increase in LDL-C and TG levels after KT. An increase in the number of patients presenting with concentric hypertrophy suggests that these metabolic changes may negatively affect cardiac structure, acting as an important risk factor for MACE compared with the overall improvement in eccentric hypertrophy after KT. Indeed, a change in RWT was significantly associated with the MACE incidence after KT. The group with increased RWT had a higher MACE, even in a multivariate analysis adjusted for classical risk factors of CVDs. Importantly, although baseline echocardiographic parameters before KT were not associated with post-KT outcomes, only changes in RWT after KT could predict MACE, indicating that changes in cardiac structure and function after KT may be more important in cardiovascular risk stratification than cardiac abnormalities before KT.

Studies conducted in patients with a hypertensive population also reported that LV geometry can be a predictor of cardiovascular morbidity and mortality. In particular, among the changes, the changes in LV geometry during antihypertensive treatment, cardiovascular morbidity, and mortality were significantly greater in concentric than in eccentric geometry.^24^ A study in the general population has shown that increased RWT and abnormal LV geometry independently predict adverse cardiovascular events, suggesting that RWT serves as an important cardiovascular risk marker across diverse patient populations.^25^ However, regarding the interaction between LV remodeling and allograft outcomes, a recent study observed that persistent LV hypertrophy was strongly associated with an increased risk of graft failure but not with cardiovascular outcomes.^13^ In our large multicenter prospective transplant cohort, we examined the effects of changes in LV geometry on KT outcomes and found that LV hypertrophy, particularly concentric hypertrophy, was linked to new-onset MACE, differing from these retrospective results. The close association between concentric geometry and MACE could be explained using features such as reduced myocardial contractility, severe diastolic dysfunction, increased risk of arrhythmias, and sudden death, although our findings cannot explain the exact mechanism of concentric geometry that may predispose patients to MACE.^26^, ^27^, ^28^

This study had some limitations. First, certain cardiovascular risk factors, such as allograft dysfunction, immunosuppressant dosage, posttransplantation complications, and economic situations, were not investigated. Second, other cardiac diseases, such as pulmonary hypertension, valvular heart disease, and arrhythmia, were not evaluated, although various echocardiographic parameters were analyzed. Third, our study included only patients who underwent both baseline and follow-up echocardiography (600 out of 1,080 total KNOW-KT participants), which may introduce selection bias as patients without follow-up echocardiography could have different clinical characteristics, disease severity, or outcomes. Fourth, the absence of detailed information on steroid dosing and rejection treatment protocols limited our ability to assess their potential impact on metabolic complications and cardiac remodeling. Finally, the participants were mainly from Korea, and hence, the results may not apply to other racial groups.

## Conclusion

This large-scale multicenter prospective study evaluated the changes in metabolic and hemodynamic burden and cardiac structure and function for more than 3 years. Although KT improved LV structural parameters overall, the relatively greater improvement in LV chamber size compared with wall thickness resulted in increased RWT. Hemodynamic improvements were associated with decreased LV size, whereas better metabolic control was associated with greater wall thickness improvement. An increase in RWT independently predicted MACE occurrence. These findings suggest that optimizing metabolic control to promote more balanced LV structural improvement could enhance cardiovascular outcomes in patients receiving KTs, highlighting the importance of comprehensive metabolic management in posttransplant care.
