# Supplementary material for: Longitudinal Changes in Left Ventricular Geometry After Kidney Transplantation and Their Implications on Cardiovascular Risk
Source: Kidney Med. 2025 Dec 11;8(2):101201. doi: 10.1016/j.xkme.2025.101201 (PMC12856460; doi:10.1016/j.xkme.2025.101201)
Supplement: Supplementary File (PDF) — Tables S1-S6 [file mmc1.pdf]

**Table S1. Univariable analysis of clinical covariates for the major cardiovascular events**

| | Univariable hazard ratio (95% CI) | *P* |
|---|---|---|
| **LVEF change per 1SD** | 0.86 (0.51 ,1.45) | 0.565 |
| **LVMI change per 1SD** | 1.49 (0.84 ,2.66) | 0.176 |
| **LVEDD change per 1SD** | 0.84 (0.51 ,1.37) | 0.474 |
| **RWT change per 1SD** | 2.11 (1.27 ,3.50) | 0.004 |
| Baseline LVEF per 1SD | 0.89 (0.56 ,1.42) | 0.622 |
| Baseline LVMI per 1SD | 1.08 (0.63 ,1.86) | 0.784 |
| Baseline LVEDD per 1SD | 1.00 (0.61 ,1.66) | 0.994 |
| Baseline RWT per 1SD | 0.92 (0.54 ,1.55) | 0.739 |
| Age per 1 year | 1.09 (1.03 ,1.16) | 0.003 |
| Male | 2.49 (0.70 ,8.83) | 0.157 |
| DM | 4.44 (1.58 ,12.49) | 0.005 |
| HTN | 2.65 (0.14 ,48.54) | 0.512 |
| Dyslipidemia | 1.00 (0.36 ,2.75) | 0.997 |
| Ever smoking | 1.69 (0.60 ,4.75) | 0.319 |
| dialysis duration per 1 year | 1.01 (0.996 ,1.01) | 0.312 |
| Tacrolimus | 1.59 (0.09 ,29.14) | 0.755 |
| BMI change per 5 kg/m$^2$ | 0.56 (0.14 ,2.25) | 0.415 |
| WHR change per 0.1 | 2.15 (0.68 ,6.75) | 0.192 |
| Hb change per 5 g/dL | 1.43 (0.49 ,4.21) | 0.514 |
| eGFR change per 5 mL/min/1.732 m$^2$ | 1.06 (0.94 ,1.21) | 0.337 |
| Donor type: Living | 0.48 (0.15 ,1.50) | 0.208 |
| Donor age per 1 year | 0.99 (0.95 ,1.04) | 0.759 |
| HbA1c change per 1% | 1.12 (0.75 ,1.68) | 0.571 |
| SBP change per 1 mmHg | 1.01 (0.99 ,1.04) | 0.278 |
| DBP change per 1 mmHg | 1.01 (0.97 ,1.05) | 0.671 |
| LDL-C change per 1 mg/dL | 1.01 (0.99 ,1.02) | 0.401 |
| TG change per 1 mg/dL | 1.00 (0.996 ,1.01) | 0.712 |
| HDL-C change per 1 mg/dL | 1.00 (0.97 ,1.04) | 0.895 |
| TG/HDL-C ratio change | 1.03 (0.88 ,1.21) | 0.719 |

Abbreviations: BMI = body mass index; DBP = diastolic blood pressure; DM = diabetes mellitus; eGFR = estimated glomerular filtration rate; Hb = hemoglobin; HbA1C = glycosylated hemoglobin; HDL-C = low-density lipoprotein cholesterol; HTN = hypertension; LDL-C = low-density lipoprotein cholesterol;

LVEDD = left ventricular end-diastolic dimension; LVEF = left ventricular ejection fraction; LVMI = left ventricular mass index; SBP = systolic blood pressure; RWT = right wall thickness; SD = standard deviation; TG = triglyceride; WHR = waist-to-hip ratio.

**Table S2. Baseline characteristics of the study population**

| | Mean ± SD or n (%) | |
|---|---|---|
| Age (years) | 46.2 | ± 11.4 |
| Men | 372 | (62.0) |
| Ever-smoker | 288 | (48.2) |
| Cause of CKD | | |
| DM | 128 | (21.3) |
| HTN | 122 | (20.3) |
| GN | 188 | (31.3) |
| PCKD | 34 | (5.7) |
| Other | 32 | (5.3) |
| Unknown | 96 | (16.0) |
| Pre-Tx. Renal replacement therapy | | |
| Pre-emptive transplantation | 138 | (23.0) |
| Hemodialysis | 381 | (63.5) |
| Peritoneal dialysis | 81 | (13.5) |
| Dialysis duration, median (IQR) | 4 (1–26) | |
| Only Dialysis duration > 0 | 10 (2–38) | |
| Donor age (years) | 44.2 | ± 11.9 |
| Donor type | | |
| Living | 512 | (85.3) |
| Living related | 313 | (52.2) |
| Living unrelated | 199 | (33.2) |
| Deceased | 88 | (14.7) |
| Immunosuppressants | | |
| Tacrolimus | 571 | (95.2) |
| Cyclosporin | 27 | (4.5) |

Abbreviations: CKD = chronic kidney disease; DM = diabetes mellitus; GN = glomerulonephritis; HTN = hypertension; IQR = interquartile range; PCKD = polycystic kidney disease; SD = standard

deviation.

**Table S3. Changes of cardiac index between baseline and follow-up**

| Mean ± SD or n (%) | Baseline (N=600) | At 3 years (N=600) | At 5 years (N=331) | *P*[a] | *P*[b] |
|---|---|---|---|---|---|
| LVPWT | 10.0 ± 1.6 | 9.5 ± 1.5 | 9.6 ± 1.4 | <.001 | <.001 |
| LVEDD (mm) | 51.2 ± 5.6 | 47.1 ± 4.8 | 46.4 ± 4.7 | <.001 | <.001 |
| LVEF (%) | 61.4 ± 7.9 | 64.8 ± 6.0 | 64.7 ± 6.8 | <.001 | <.001 |
| IVSWT | 10.0 ± 1.6 | 9.7 ± 1.5 | 9.8 ± 1.6 | <.001 | 0.029 |
| RWT | 0.39 ± 0.07 | 0.41 ± 0.07 | 0.42 ± 0.07 | <.001 | <.001 |
| RWT>0.42 | 179 (29.8) | 218 (36.5) | 135 (40.8) | 0.003 | 0.046 |
| LAD | 39.2 ± 6.7 | 37.4 ± 6.2 | 37.4 ± 6.0 | <.001 | <.001 |
| RWMA | 31 (5.3) | 22 (3.8) | 9 (2.8) | 0.160 | 0.405 |
| LVESD | 33.6 ± 6.1 | 29.9 ± 4.7 | 29.2 ± 4.5 | <.001 | <.001 |
| LVMI | 113.4 ± 31.8 | 94.4 ± 23.4 | 93.6 ± 23.1 | <.001 | <.001 |
| Valvular calcification | 72 (12.2) | 87 (16.3) | 81 (18.3) | 0.001 | 0.003 |
| Calculated LVMI | 113.6 ± 30.9 | 93.0 ± 22.9 | 91.0 ± 21.8 | <.001 | <.001 |
| LV geometry | | | | <.001 | <.001 |
| Normal | 215 (35.8) | 304 (51.2) | 168 (50.9) | | |
| LVCR | 64 (10.7) | 147 (24.7) | 87 (26.4) | | |
| LVCH | 115 (19.2) | 70 (11.8) | 47 (14.2) | | |
| LVEH | 206 (34.3) | 73 (12.3) | 28 (8.5) | | |

Abbreviations: DBP = diastolic blood pressure; LAD = left atrial diameter; LVCH = left ventricular concentric hypertrophy; LVCR = left ventricular concentric remodeling; LVEDD = left ventricular end-diastolic dimension; LVEF = left ventricular ejection fraction; LVEH = left ventricular eccentric hypertrophy; LVESD = left ventricular end-systolic dimension; LVMI = left ventricular mass index; LVPWT = left ventricular posterior wall thickness; IVST = interventricular septum thickness; RWMA = regional wall motion abnormality; RWT = right wall thickness; SBP = systolic blood pressure; SD = standard deviation;

[a]*P*-value for comparison between baseline and 3 years

[b]*P*-value for comparison between baseline and 5 years

**Table S4. Correlation between echocardiographic parameters and clinical data**

| Changes of echocardiographic parameters | | Changes from baseline to 3 years | | | | | | |
|---|---|---|---|---|---|---|---|---|
| | | Hemoglobin | SBP | eGFR | HbA1c | TG | HDL-cholesterol | TG/ HDL-cholesterol Ratio |
| | Mean ± SD | 3.4 ± 2.4 | -9.9 ± 23.3 | 0.2 ± 21.0 | 0.8 ± 1.2 | 11.4 ± 90.4 | 15.0 ± 15.7 | -0.7 ± 3.0 |
| | | r (*P*) | r (*P*) | r (*P*) | r (*P*) | r (*P*) | r (*P*) | r (*P*) |
| LVEDD | -4.1 ± 5.4 | -0.166 (<.001) | 0.225 (<.001) | 0.032 (0.438) | -0.12 (0.006) | -0.162 (<.001) | 0.057 (0.173) | -0.147 (<.001) |
| LWEF | 3.4 ± 7.4 | -0.048 (0.241) | -0.062 (0.131) | -0.105 (0.01) | 0.035 (0.422) | 0.056 (0.175) | -0.03 (0.469) | 0.073 (0.08) |
| RWT | 0.0 ± 0.1 | 0.074 (0.072) | -0.034 (0.417) | -0.079 (0.055) | 0.096 (0.027) | 0.133 (0.001) | -0.008 (0.847) | 0.116 (0.005) |
| LVMI | -18.3 ± 30.9 | -0.159 (<.001) | 0.291 (<.001) | -0.029 (0.509) | -0.036 (0.455) | -0.067 (0.135) | 0.06 (0.184) | -0.075 (0.096) |

Abbreviations: eGFR = estimated glomerular filtration rate; HbA1C = glycosylated hemoglobin; HDL-C = low-density lipoprotein cholesterol; LVEDD = left ventricular end-diastolic dimension; LVEF = left ventricular ejection fraction; LVMI = left ventricular mass index; r = Pearson's correlation coefficient; RWT = right wall thickness; SBP = systolic blood pressure; SD = standard deviation; TG = triglyceride levels.

**Table S5. Impact of baseline echocardiographic parameters on the major cardiovascular events (MACE)**

| | Cumulative incidence (95% CI) (%) | HR (95% CI) | *P* |
|---|---|---|---|
| MACE | 5.18 (3.65–7.33) | | |
| EF per 1SD | | 0.98 (0.69 , 1.39) | 0.904 |
| LVMI per 1SD | | 1.05 (0.73 , 1.52) | 0.781 |
| LVEDD per 1SD | | 1.00 (0.70 , 1.43) | 0.990 |
| RWT per 1SD | | 1.00 (0.70 , 1.43) | 0.990 |
| IVST per 1SD | | 1.09 (0.77 , 1.55) | 0.615 |
| LVPWT per 1SD | | 1.00 (0.70 , 1.43) | 0.980 |

Abbreviations: CI, confidence interval; EF, ejection fraction; HR, hazard ratio; IVST, interventricular septum thickness; LVEDD, left ventricular end-diastolic dimension; LVMI, left ventricular mass index; LVPWT, left ventricular posterior wall thickness; RWT, relative wall thickness

**Table S6. Clinical outcome by change in echocardiographic parameters after 3-year follow-up**

| | Death | Cardiac event | Stroke | MACE | Graft loss |
|---|---|---|---|---|---|
| | Cumulative incidence (95% CI) (%) | Cumulative incidence (95% CI) (%) | Cumulative incidence (95% CI) (%) | Cumulative incidence (95% CI) (%) | Cumulative incidence (95% CI) (%) |
| Overall | 0.96 (0.40–2.29) | 1.99 (1.11–3.57) | 0.53 (0.17–1.62) | 2.71 (1.64–4.45) | 2.61 (1.56–4.37) |
| LVEF change | | | | | |
| 1st Q | – | 0.71 (0.10–4.96) | – | 0.71 (0.1–4.96) | 1.57 (0.40–6.15) |
| 2nd Q | 3.31 (1.25–8.57) | 2.36 (0.76–7.18) | 2.18 (0.70–6.65) | 4.52 (2.04–9.83) | 4.03 (1.70–9.42) |
| 3rd Q | – | 3.06 (1.28–7.22) | – | 3.72 (1.69–8.1) | 1.92 (0.62–5.84) |
| 4th Q | 0.83 (0.12–5.72) | 1.67 (0.42–6.45) | – | 1.65 (0.42–6.45) | 3.11 (1.18–8.09) |
| Log-rank *P* | 0.175 | 0.523 | – | 0.181 | 0.568 |
| LVMI change | | | | | |

| | | | | | |
|---|---|---|---|---|---|
| 1st Q | – | 0.88 (0.12–6.06) | 0.79 (0.11–5.46) | 1.66 (0.42–6.48) | 2.56 (0.83–7.74) |
| 2nd Q | – | 1.57 (0.40–6.15) | – | 1.57 (0.4–6.15) | 0.89 (0.13–6.17) |
| 3rd Q | 0.89 (0.13–6.17) | 2.48 (0.80–7.51) | – | 2.48 (0.8–7.51) | 2.65 (0.86–8.00) |
| 4th Q | 1.83 (0.46–7.14) | 2.64 (0.86–7.99) | 1.71 (0.43–6.68) | 4.33 (1.82–10.13) | 4.42 (1.87–10.30) |
| Log-rank *P* | 0.550 | 0.732 | 0.555 | 0.551 | 0.435 |
| LVEDD change | | | | | |
| 1st Q | 0.78 (0.11–5.42) | 2.94 (1.11–7.67) | 0.69 (0.10–4.79) | 3.62 (1.52–8.5) | 2.30 (0.75–6.95) |
| 2nd Q | 0.71 (0.10–4.96) | 2.07 (0.67–6.30) | 0.71 (0.10–4.96) | 2.78 (1.05–7.25) | 3.50 (1.47–8.20) |
| 3rd Q | 0.77 (0.11–5.33) | 1.44 (0.36–5.66) | 0.67 (0.09–4.67) | 2.11 (0.68–6.43) | 2.26 (0.74–6.83) |
| 4th Q | 1.63 (0.41–6.34) | 1.49 (0.37–5.88) | – | 2.32 (0.75–7.06) | 2.27 (0.74–6.91) |
| Log-rank *P* | 0.860 | 0.793 | 0.998 | 0.858 | 0.900 |
| RWT change | | | | | |
| 1st Q | 1.55 (0.39–6.06) | 0.78 (0.11–5.42) | – | 0.78 (0.11–5.37) | 2.29 (0.74–6.93) |

| | | | | | |
|---|---|---|---|---|---|
| 2nd Q | 0.78 (0.11–5.22) | 0.67 (0.09–4.64) | – | 0.67 (0.09–4.64) | 0.75 (0.11–5.18) |
| 3rd Q | 0.78 (0.11–5.37) | 2.89 (1.09–7.53) | 0.67 (0.09–4.64) | 4.33 (1.96–9.42) | 1.44 (0.36–5.64) |
| 4th Q | 0.78 (0.11–5.42) | 3.71 (1.56–8.71) | 1.45 (0.36–5.68) | 5.12 (2.47–10.47) | 5.97 (3.03–11.58) |
| Log-rank *P* | 0.892 | 0.187 | 0.560 | 0.037 | 0.038 |

Abbreviations: CI, confidence interval; LVEDD, left ventricular end-diastolic dimension; LVEF, left ventricular ejection fraction; LVMI, left ventricular mass index; MACE, Q, quartile; RWT, relative wall thickness
